# The frequency of spontaneous triploidy in farmed Atlantic salmon produced in Norway during the period 2007–2014

**DOI:** 10.1186/s12863-015-0193-0

**Published:** 2015-04-11

**Authors:** Kevin A Glover, Abdullah S Madhun, Geir Dahle, Anne G E Sørvik, Vidar Wennevik, Øystein Skaala, H Craig Morton, Tom J Hansen, Per G Fjelldal

**Affiliations:** Institute of Marine Research, PO Box 1870, Nordnes 5817 Bergen, Norway

**Keywords:** Autopolyploidy, Autotriploid, Microsatellite, Breeding, Aquaculture, Escapees, genetic

## Abstract

**Background:**

Spontaneous triploidy has been reported in a number of fish species, and is often linked with *in vivo* or *in vitro* ageing of eggs post ovulation. Here, we provide the first investigation into the frequency of spontaneous triploidy in farmed Atlantic salmon by analysing more than 4000 fish from 55 farms, and approximately 1000 recaptured escapees, all sampled in the period 2007–2014. In addition, we compare microsatellite genotyping against flow cytometry and red blood cell diameter in a set of 45 putatively diploid and 45 putatively triploid Atlantic salmon.

**Results:**

The three methods implemented for ploidy determination gave consistent results, thus validating the methods used here. Overall, 2.0% spontaneous triploids were observed in salmon sampled on farms. The frequency of spontaneous triploids varied greatly among sea cages (0-28%), but they were observed in similar frequencies among the three primary breeding companies (1.8-2.4%). Spontaneous triploids were observed in all farming regions in Norway, and in all years sampled. Spontaneous triploids were also observed among the escapees recaptured in both the marine environment and in rivers.

**Conclusions:**

Spontaneous triploidy in commercially produced Atlantic salmon is likely to be a result of the practices employed by the industry. For logistical reasons, there is sometimes a pause of hours, and in some cases overnight, between killing the female broodfish, removal of her eggs, and fertilization. This gives the eggs time to age post ovulation, and increases the probability of duplication of the maternal chromosome set by inhibition of the second polar body release after normal meiosis II in the oocyte.

**Electronic supplementary material:**

The online version of this article (doi:10.1186/s12863-015-0193-0) contains supplementary material, which is available to authorized users.

## Background

The Atlantic salmon (*Salmo salar*) farming industry was first initiated in Norway in the late 1960’s, and has now grown to become an economically significant industry in several countries. Current global production exceeds 2 million tons, and primarily involves rearing domesticated strains that have been selected for a range of commercially important traits for up to 10 generations or more [1]. A number of breeding programs for Atlantic salmon exist, for example in Norway [1-3], Scotland [4,5], Atlantic Canada [6], British Colombia Canada [7] and Australia [8]. However, as Norway is the world’s largest salmon producing nation, and genetic material from the three primary Norwegian breeders (Aqua Gen AS, Marine Harvest and Salmobreed AS) has been distributed to fish farms in other regions of the globe (e.g., [9]), farmed salmon originating from Norwegian breeding programs dominate global production.

Each year, thousands or hundreds of thousands of farmed fish escape from their net pens into the wild. While many of these escapees disappear never to be seen again, some return to freshwater and can interbreed with wild salmon [10,11]. As a result of interbreeding, genetic changes in some wild salmon populations have been reported [12-16]. In response to requests from the Norwegian Directorate of Fisheries (NDF), who are responsible for production and implementation of aquaculture regulations in Norway, the Institute of Marine Research (IMR) developed a genetic method to identify the farm of origin for farmed escapees. The method, which was named the “stand-by method”, was initially produced for the identification of salmon escapees back to their farm(s) of origin [17]. However, it has subsequently been adapted to identify the farm of origin for Atlantic cod (*Gadus morhua*) [18] and rainbow trout (*Oncorhynchus mykiss*) escapees also [19]. In short, the method works by taking samples of salmon from cages on farms in the vicinity of the recaptured escapees (termed the baseline samples = potential source of the escapees), and matches the multi locus genetic profile of each recaptured escapee to each of the baseline samples using a range of complimentary genetic assignment methods.

In the period 2007–2014, the “stand-by method” was implemented to identify the farm of origin for escaped salmon in >12 episodes [20-25], some of which have resulted in legal cases [22]. In some of these cases, the occasional triploid farmed salmon has been detected based upon its multi locus microsatellite genetic profile (i.e., displayed three alleles at multiple loci). While the Atlantic salmon aquaculture industry is currently conducting research into the potential use of triploid salmon for commercial production [26-29], during the period in which samples from this study were collected, there was either no or next to no commercial production of triploid salmon in Norway, and this only occurred on a very low number of farms. This has been confirmed by the three principal breeding companies in Norway (see acknowledgements). Thus, these observed triploid salmon arose as a result of a spontaneous event and not a deliberate protocol.

Spontaneous triploidy is a phenomena that has been observed in a number of fish species [30], for example rainbow trout [31], tench (*Tinca tinca*) [32-34], Japanese eel (*Anguilla japonica*) [35], coho salmon (*Oncorhynchus kisutch*) [36], European catfish (*Silurus glanis*) [37], and sterlet (*Acipenser ruthenus*) [38]. Within tetraploid fish, such as the Siberian sturgeon (*Acipenser baerii*), spontaneous hexaploidy, which involves the same mechanism as spontaneous triploidy, has also been observed [39]. While spontaneous triploidy has been observed in natural populations [30], it is primarily documented in cultured and farmed populations. Spontaneous triploidy has also been reported in cultured Atlantic salmon [40,41]. However, the extent and frequency of this phenomenon in commercial Atlantic salmon farming has yet to be studied. Here, based upon the re-analysis of an extensive database of genetic data for farmed salmon collected in association with the “stand-by method”, we investigate the frequency of spontaneously occurring triploid salmon in commercial farms in Norway in the period 2007–2014.

## Methods

### Samples

**Figure 1:**
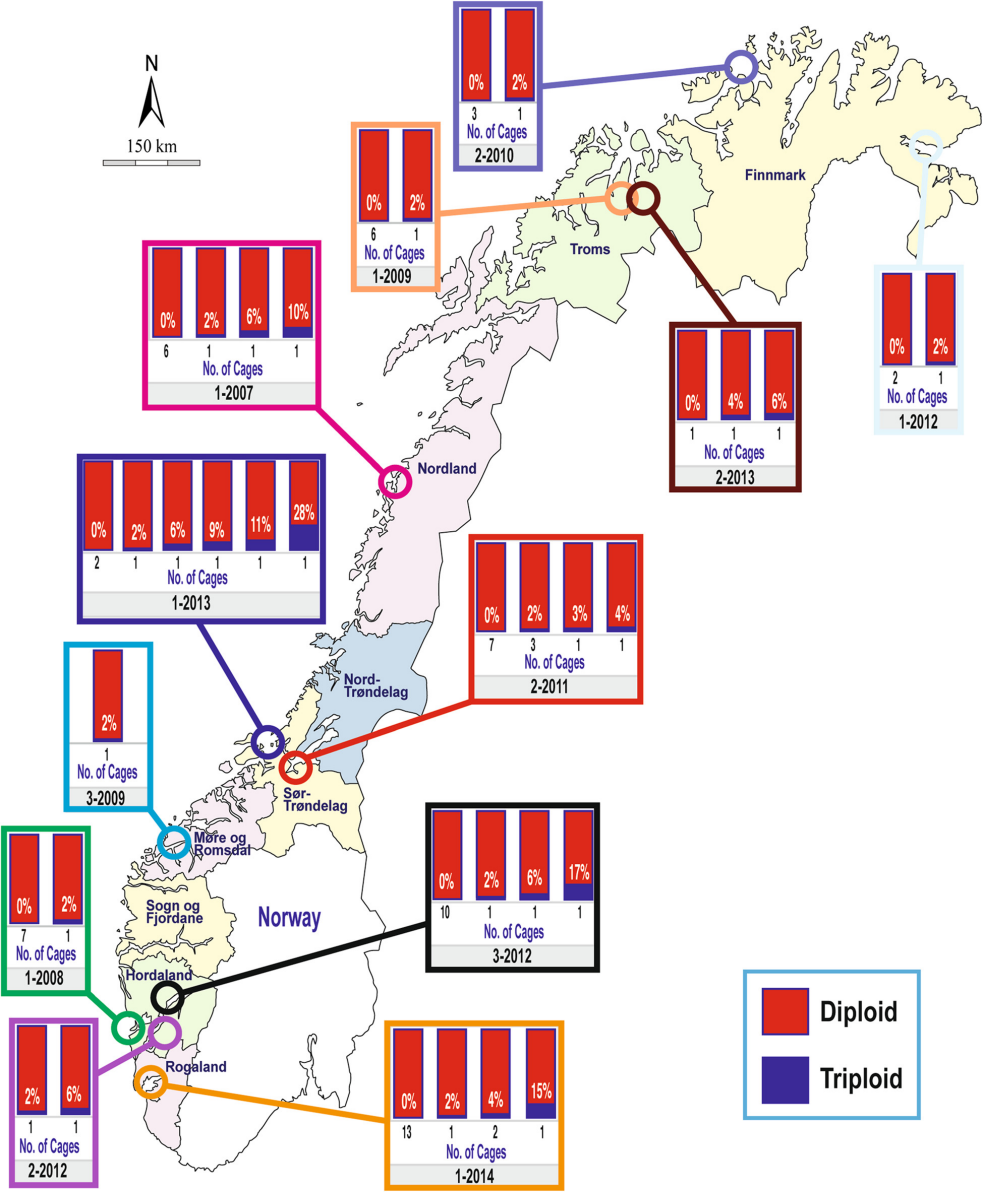
The observed frequencies of spontaneous tripoloid salmon sampled from 86 marine cages located on 55 marine farms in Norway in the period 2007–2014. Colour is only used for visual enhancement.

**Figure 2:**
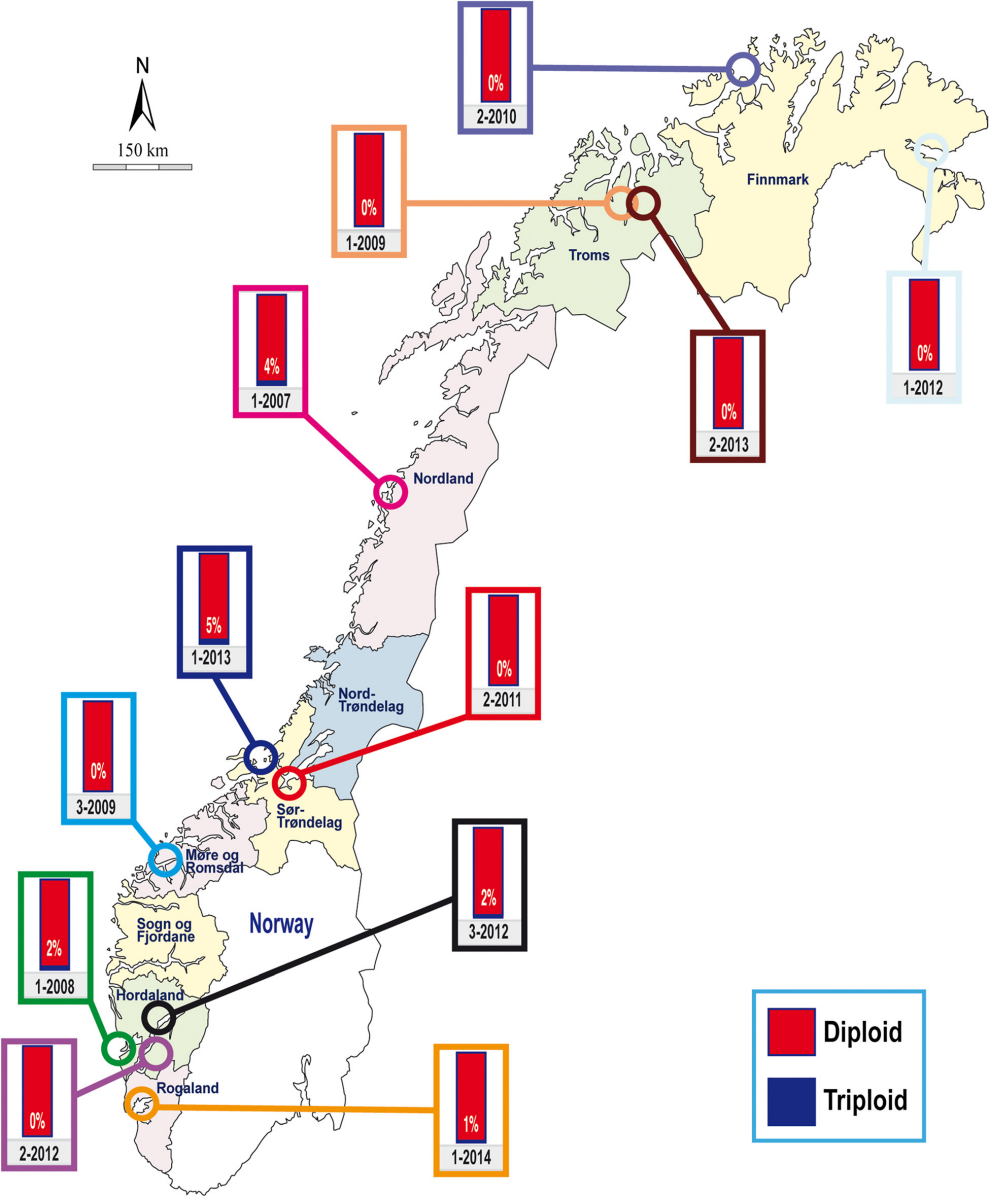
The observed frequencies of spontaneous triploid salmon farmed escapees recaptured in Norway in the period 2007–2014. Colour is only used for visual enhancement.

The present study is based upon the analysis of 5051 farmed salmon. Of these samples, 4089 were collected from 86 fish-cages located on 55 Norwegian commercial salmon farms spanning the entire Norwegian coastline (Figure 1, Additional file 1), while the remaining 962 salmon were sampled as escapees in both fresh and saltwater (Figure 2, Additional file 1). All of these samples were taken in association with the DNA stand-by method in the period 2007–2014. For legal reasons, only the approximate locations of the farms and the recapture sites of the escapees are revealed (Figures 1 and 2). As the primary objective of the study was to identify the frequency of spontaneous triploidy in farmed Norwegian salmon, precise farm information is not of importance.

From each farm, adipose fin clips were randomly sampled from approximately 45–47 salmon (range 41–90) from a single cage using a wet net. The samples were thereafter placed into a single tube containing alcohol for preservation. No phenotypic nor rearing information was taken from these fish nor the farms from which they were sampled. These tissue samples were initially used to create a baseline genetic profile of the cage of fish for genetic assignment analysis [22]. Multiple cages were sampled on the same farm in cases where the farm had salmon originating from more than one juvenile or smolt producer, and therefore potentially contained fish of more than 1 genetic origin. In addition to the samples originating from fish farms, farmed escapees, originating from the same regions as the farms themselves (Figure 2) were collected. The numbers of farmed escapees varied between 16 and 343 per data set (Additional file 1).

Tissue samples of salmon from commercial farms were taken by employees of the Norwegian Directorate of Fisheries together with the farmer owning the fish. Tissue samples of the farmed escapees were taken from fish that had been already captured and killed as a result of recreational angling or netting. The researchers of this research played no part in the sampling conducted. Consequently, specific permits for collection of these samples were not required by Norwegian or international law for the use of animals in research.

### Genotyping

Prior to genetic analysis, all samples were organized into a database which allocates a unique number for all samples which includes its initial tissue tube and subsequent DNA isolation plate number. Blind genotyping control is routinely conducted in this laboratory [21,42,43]. Although the frequency of blind re-genotyping controls varied between the data sets included in this analysis, in all cases, a number of randomly selected fish had their DNA isolated twice in order to conduct blind genotyping and sample control. This equated to a minimum of 10% of the samples included in this study (>500 samples re-analysed). Genotyping error rates for parts of the data sets included in this study have been published previously [21], and for other data sets using the same markers in this laboratory [13]. In all cases, the genotyping error rates are very low, and are almost exclusively associated with homozygote and heterozygote errors. Thus far, no mis-identification between triploid nor diploid offspring have been detected, also when the pedigree of the parents and triploid offspring from single families has been double checked using parentage testing approaches [40].

DNA was isolated in 96 well format using the DNeasy blood and tissue kit from Qiagen. Each plate contained at least 2 blank cells as negative controls. As these analyses were conducted over an extended period of time, the exact protocols used for PCR amplification varied, however, the latest laboratory protocols for amplification of the microsatellite markers used here are available upon request. For each sample, between 15–18 microsatellite loci were amplified. These included the following loci organized into three multiplex reactions: *SSsp3016* (Genbank no. AY372820), *SSsp2210*, *SSspG7, SSsp2201, SSsp1605, SSsp2216* [44], *Ssa197, Ssa171, Ssa202* [45], *SsaD157, SsaD486, SsaD144* [46], *Ssa289, Ssa14* [47], *SsaF43* [48], *SsaOsl85* [49], *MHC I* [50] *MHC II* [51]. PCR products were analysed on an ABI 3730 Genetic Analyser and sized by a 500LIZ™ size-standard. Automatically binned alleles were manually checked by two researchers prior to exporting data for statistical analyses.

### Protocol for triploid identification using microsatellites

Triploid organisms are readily identified through microsatellite DNA genotyping as they often display three clearly identifiable alleles per locus [52] (Additional file 1). However, not all loci will display trisomy in a triploid individual. This depends upon the genotype of the mother and the father, and the distance of the given microsatellite locus from the centromere, which is in turn linked with the probability of crossing over.

In order to provide a conservative estimate of the frequency of triploidy in the present study, we only reported an individual as triploid in the case that it displayed three clear alleles at two or more of the 15–18 loci genotyped. Individual salmon displaying three alleles at only one locus were not reported as triploid (this only occurred for two individuals and thus does not influence the results of the present study). Thus, the approach we have implemented to identify the frequency of triploids is identical to a recent study documenting spontaneous triploids in cultured Atlantic salmon in a Baltic fish hatchery [41], and is similar to the approach used to identify triploids in a variety of other fish and insect studies of triploidy [52-55]. Finally, these genetic markers in this laboratory have been used to extensively conduct parentage testing for Atlantic salmon in common-garden experiments [40,56-58]. In one of these parentage-based studies, triploid salmon offspring were identified in the pedigree. All of these individuals were re-genotyped and accurately re-identified a second time using these markers demonstrating the reliability of the approach to consequently differentiate between diploid and triploid individuals [40].

### Validation of microsatellite genotyping for triploid identification

Microsatellite genotyping for triploid identification is an established technique (see above), and has also been validated against other triploid identification methods in species such as the *Carassius auratus* complex [59], and turbot (*Scophthalmus maximus*) [52]. Nevertheless, we validated microsatellite genotyping against a set of 45 putatively diploid and 45 putatively triploid farmed salmon samples in the present study using red blood cell (RBC) diameter measurements and flow cytometry which estimates DNA content. The putatively triploid salmon used for this validation had been produced by a deliberate pressure shock treatment. All of these fish had been killed by a sharp blow to the head prior to taking samples for analysis.

For each putatively diploid and putatively triploid salmon used in the ploidy method validation, fin clips for DNA analysis, and blood was collected. From each sample, 10 μl heparinised blood was fixed for 30–60 min with 4% PFA. The blood cells were washed three times in PBS, and re-suspended in 300 μl PBS containing 50 ug/ml propidium iodide (Life Technologies), 50 μg/ml Rnase A (Life Technologies), and 0.3% (v/v) Tween-20 (Sigma), and kept at room temperature overnight in the dark. Next day the cells were analyzed on a FACSCanto II flow cytometer (BD Biosciences). Cell debris and doublets were eliminated from the analysis by gating, and the data were analyzed using BD FACSDIVA software. The average diameter of the salmon RBCs were measured from blood smears (IMAGEPRO PLUS, version 4.0; Media Cybernetics, Silver Spring, MD). Ten erythrocytes per fish were measured. Microsatellites were genotyped according to the same protocols as described for the main data set above. Once data was produced using all three methods, ploidy results were compared.

## Results

### Validation of ploidy determination method

In total, 81 of the 90 samples used for method validation had their ploidy determined by all three methods, with the remaining 9 samples identified by two of the methods (Additional file 2). There was complete agreement between the identification of ploidy using all three approaches. A single triploid salmon was identified in the putatively diploid group of salmon, while 11 diploid salmon were identified in the putatively triploid group of salmon. Thus, microsatellites can be used to reliably determine ploidy in Atlantic salmon where blood samples are not available, as in the main part of the current study.

### General trends in samples from cages and escapees

Of the 5051 farmed salmon analysed in the period 2007–2014, a total of 91 (1.8%) spontaneously arising triploids were detected (Figure 1, Figure 2, Table 1). The number of loci displaying two or three alleles for each of the 91 spontaneous triploid salmon is presented in table form, as is a graphical representation of the profiles for a single diploid and triploid salmon example (Additional file 1).

**Table 1:** **The observed frequencies of diploid and spontaneous triploid salmon according to sampling source and genetic background**

**Data source**	**Diploid (n)**	**Triploid (n)**	**Total (n)**	**% Triploid**
Samples from farms				
Aqua Gen AS	1635	37	1672	2.2
Salmobreed	783	19	802	2.4
Mowi	609	11	620	1.8
Unknown	1062	15	1077	1.4
Farms total	4089	82	4171	2.0
Samples of recaptured escapees				
Recaptured freshwater	376	4	380	1.1
Recaptured saltwater	586	5	591	0.8
Escapees total	962	9	971	0.9
All samples combined	5051	91	5142	1.8

Note, escapees are not assigned to genetic strain and therefore represent an unquantified mixture of fish from all strains. Fish of “unknown” strain sampled on commercial farms represent an undocumented mixture of all strains.

Samples collected in the period 2007–2014.

Triploids were observed in all regions in Norway, and across the entire time period. Triploids were observed among the fish sampled in cages, as well as among the farmed escapees recaptured in the wild.

### Samples from fish cages

Looking specifically at the samples collected from the 86 cages located on 55 farms, the frequency of spontaneous triploidy varied greatly (Figure 1). In most cages sampled, no triploid salmon were observed. In a few cages, a low frequency, which typically meant 1–3 individuals from 45–47 fish typically sampled (Additional file 1), were detected (i.e., 2-5%). In five of the cages sampled, the reported frequency of triploid salmon ranged from 10-28%. These five cages displaying notably higher frequencies of spontaneous triploidy, originated from separate case studies, and were therefore not connected to any single farm (Figure 1, Additional file 1).

In many, but not all of the cages where samples were taken, the genetic breeding line of the salmon was unequivocally determined (based upon document information collected by the NDF when the samples were taken on each farm). When the triploid frequency data was split into the three main breeding companies producing salmon in Norway, these data revealed that triploids were observed in all three breeding programs, and notably, that the observed frequency of spontaneous triploidy was similar among strains (Table 1). Among the salmon of unidentified breeding origin collected from fish farms (i.e., the paperwork associated with the sample was not precise enough to unequivocally determine the genetic strain), the frequency of triploid salmon was slightly lower, but still close to the average numbers (Table 1).

### Samples of escapees

Overall, the frequency of triploid salmon among the escapees was 0.9% which is lower than the observed frequencies for the samples taken in cages (Table 1). Triploid salmon escapees were observed among the recaptured escapees in 6 of the 12 cases investigated, and in different regions (Figure 2). Most of the escapees were sampled in salt water (Table 1), however, the observed frequency of triploids recaptured in freshwater was similar to the observed frequency recaptured in salt water.

## Discussion

This study represents the first systematic investigation into the frequency of spontaneous triploidy in farmed Atlantic salmon. The genetic analysis of more than 5000 salmon collected in the period 2007–2014 gave the following main results: 1. The overall observed frequency of spontaneous triploidy among salmon collected from 55 farms was 2.0%, 2. Spontaneous triploidy occurred in farmed salmon originating from all three major breeding lines in Norway, and in similar frequencies, 3. Spontaneous triploidy was observed in farms located in all regions of Norway, and in all years sampled spanning from 2007 to 2014, 4. The frequency of spontaneous triploidy varied greatly among cages (ranging 0-28%), 5. Spontaneous triploids were observed amongst the escapees recaptured in both freshwater and the marine environment, 6. The validation tests implemented here demonstrated that microsatellite genotyping gives consistent results for ploidy determination as RBD diameter measurements and flow cytometry in this species.

Based upon personal communications with the three primary breeding companies operating in Norway, we were able to exclude the possibility that the triploid salmon observed in this study were the result of a deliberate pressure shock protocol to produce triploid fish as is used in Atlantic salmon experiments [26-28]. Thus, the reported triploids arose as a result of spontaneous event, as has been observed previously for Atlantic salmon [40,41], and a range of other fish species in culture [31-33,35,36], and in the wild [30]. While the Atlantic salmon farming industry has very recently started experimental production of triploid salmon in Norway [26-29], for the timescale in which the samples in this study were collected, very few triploid salmon were commercially produced. Furthermore, in all cases where triploid salmon were deliberately produced by these farming companies, triploids were sold to a very limited number of farms in specific locations. It has been verified by these three companies that none of these locations nor farms overlapped with the farms from which samples upon which the present study is based. While it is still theoretically possible that some of the recaptured triploid escapees could have arisen from a farm(s) outside of the sampling regions in the present analyses, and therefore from a farm that reared deliberately produced triploids, this remote possibility is highly unlikely given the fact that the vast majority of the escapees analysed in the present study had already been assigned to the farms sampled here based upon their genetic profiles [20-24]. Furthermore, the observed frequency in the escapees was lower than from the farms with a documented background, and has therefore not spuriously contributed to an inflated estimate of spontaneous triploid frequency within the industry as a whole.

Spontaneous triploidy originates from the duplication of the maternal chromosome set by inhibition of the second polar body release after normal meiosis II (crossing over) in the oocyte. As a result of observing the non-random distribution of this phenomena, it has been suggested that it may have an underlying genetic basis or predisposition [60]. This is supported by positive heritability estimates for propensity of this phenomena in common carp (*Cyprinus carpio*) [61], and evidence for a paternal contribution to autopolyploidy in white sturgeon (*Acipenser transmontanus*) [62]. However, in other studies, the frequency of spontaneous triploidy has been clearly linked with *in vitro* or *in vivo* post ovulatory aging of eggs prior to fertilisation [31,33,35], and is further enhanced by increased temperatures during aging [31,33]. Incidentally, the eggs used in the Atlantic salmon experiment by [40], where spontaneous triploids were observed in the resulting offspring, had been transported approximately 6–8 hours by car prior to fertilisation. It appears possible that the delayed fertilization combined with possible temperature increase in the car during transport could have been the trigger for the observed spontaneous triploidy in that case [40].

Turning attentions back to the observations in the present study, it is important to note that spontaneous triploidy was reported in all three breeding companies, and at similar frequencies (Table 1). This strongly suggests that the genetic material produced by all three of these companies, which account for most of the farmed salmon produced globally, display similar propensities for this phenomena. Furthermore, due to the logistics of the breeding practices on commercial farms, eggs are sometimes removed from female broodfish up to several hours after they have been killed. In addition, once the eggs have been removed from the female broodfish, they may be stored further for several hours or even until the next day before they are fertilised. Given the fact that storage and aging of eggs post ovulation has been documented to increase the frequency of spontaneous triploidy [31,33,35,36,63], it is concluded that stripping practices often implemented by the Atlantic salmon aquaculture industry, with pauses between killing the broodfish and fertilsation of eggs, is the most likely explanation for the observed frequency of 2.0% spontaneous triploidy in Norwegian farmed salmon in the period 2007–2014. This conclusion is consistent with the fact that spontaneous triploidy varied greatly among the cages and farms sampled here (Figure 1), which may in turn reflect differences in treatment of unfertilized eggs for the fish reared in those cages. Controlled experiments using aged eggs could help identify the underlying mechanism(s) driving this process specifically within the Atlantic salmon aquaculture industry.

Farmed escaped salmon have successfully interbred and caused genetic changes in a number of wild Atlantic salmon populations inhabiting rivers in Norway and Ireland [12-16,64]. Thus, the Atlantic salmon farming industry is currently investigating the potential to deliberately produce triploid salmon in order to mitigate potential genetic impacts of escapees on native wild populations. As a consequence of this, the numbers of deliberately produced triploid salmon in commercial fish farms is likely to expand within the near future in both Norway and other countries. Triploid salmon are sterile, and will therefore not be able to hybridise with wild populations. Nevertheless, in a recent experiment conducted in semi-natural spawning arenas, deliberately produced triploid farmed male salmon displayed wild salmon spawning behaviors, and importantly, managed to coax a wild female salmon to spawn [65]. Thus, the potential for an ecological interaction between triploid (sterile) escapees and wild salmon, through mate competition and non-productive spawning exists. However, in order for this to occur, triploid farmed escapees first need to migrate to freshwater where wild salmon spawn. Here, we demonstrate for the first time, that spontaneous triploid salmon, escaping from commercial fish farms, can enter freshwater. Furthermore, within this study, the frequency of triploids observed among the escapees recaptured in freshwater was similar to the frequency observed among the escapees recaptured in salt water (Table 1). Nevertheless, while these data demonstrate “proof of concept”, we urge caution in interpreting the relative frequency of this occurrence between diploid and triploid salmon. First, only a small number of the escapees investigated in the present study were captured in freshwater, and second, freshwater sampling only occurred in three of the cases investigated (Additional file 1). More extensive and representative sampling of escapees in a larger number of rivers is required in order to fully evaluate the relative frequency of this occurrence.

An experimental release study conducted with diploid and triploid salmon smolts in Ireland reported significantly lower freshwater return rates for triploid fish than their diploid counterparts [66]. The fish from that release experiment first had to survive in the marine environment, migrate to the ocean feeding grounds, and then return back to the coastline and ultimately freshwater. In the present study, almost all of the escapees were the result of larger fish (typically 1-5 kg) escaping from net pens and either being captured in the sea or a local river immediately or very shortly after escape. This is based upon the fact that the “stand-by method” is almost exclusively implemented in escape events when there is a distinct and sudden appearance of escapees in a local area [17,22]. Thus, the majority of the escapees analysed in the present study have not undergone an oceanic migration as in the smolt release experiment above, which could explain the difference in the results between these two studies. Immature escaped diploid salmon have been documented to occasionally enter freshwater soon after escape [25,67]. It is therefore possible that the triploid escapees that were reported in freshwater in the present study, have displayed a similar maladapted behavior of migrating to freshwater without any maturation as has been observed for immature diploids.

## Conclusions

This study represents the first systematic investigation into the occurrence and frequency of spontaneous triploidy in farmed Atlantic salmon. Based upon the analysis of microsatellite DNA profiles of more than 4000 salmon collected from 55 fish farms, and a group of nearly 1000 farmed escapees recaptured in the wild, all sampled in the period 2007–2014, we were able to document that spontaneous triploidy occurred at 2.0% in the samples taken on farms, and 1.8% in the total material. We also documented for the first time, that triploid farmed fish escaping from commercial farms, may enter freshwater. We suggest that spontaneous triploidy occurs in farmed Atlantic salmon due to the occasional delay between stripping eggs from female broodfish and their fertilization. This has been documented in other fish species to increase the chance of duplication of the maternal chromosome set by inhibition of the second polar body release after normal meiosis II (crossing over) in the oocyte.

In the past decade, Norwegian Atlantic salmon aquaculture has produced close to or in excess of 1 million tons of farmed salmon annually. Taking an average slaughter weight of 5 kg, and an average spontaneous triploid frequency of 2%, our analyses could suggest that as many as 400 million spontaneous triploid salmon have been produced in Norwegian Atlantic salmon farming in the past decade.

## Additional files

Additional file 1:
**List of microsatellite markers displaying two or three alleles for all 91 spontaneous triploid salmon observed in the main study.** Example of microsatellite genotype profile for a normal diploid and a spontaneous triploid salmon as detected on an ABI3700XL sequencing machine using the genotyping software Genemapper. Numbers of diploid and spontaneous triploid salmon sampled per cage and among the escapees for all cases upon which the present study is based. Additional file 2:
**Results of ploidy determination for 45 putatively diploid and 45 putatively triploid Atlantic salmon using three separate methods: microsatellite DNA analysis, red blood cell diameter and flow cytometry.** There was complete agreement between all methods on this set of validation samples.
